# Degeneration of the Sacroiliac Joint in Hip Osteoarthritis Patients: A Three-Dimensional Image Analysis

**DOI:** 10.5334/jbsr.1648

**Published:** 2019-05-23

**Authors:** Maki Asada, Daisaku Tokunaga, Yuji Arai, Ryo Oda, Hiroyoshi Fujiwara, Kei Yamada, Toshikazu Kubo

**Affiliations:** 1 Department of Orthopaedics, Kyoto Prefectural University of Medicine, JP; 2 Kyoto Prefectural Rehabilitation Hospital for the Mentally and Physically Disabled, JP; 3 Department of Radiology, Kyoto Prefectural University of Medicine, JP

**Keywords:** sacroiliac joint, computed tomography, hip osteoarthritis, vacuum phenomenon, degeneration

## Abstract

**Objective:**

The sacroiliac joint is an important source of low back pain and may be influenced by pathologies in adjoining structures such as the hip or the spine. This study aimed to investigate the influence of hip osteoarthritis on sacroiliac joint degeneration by examining the sacroiliac joints of hip osteoarthritis patients, focusing on the localization and quantity of vacuum phenomena.

**Materials and Methods:**

The preoperative computed tomography (CT) of 31 female hip replacement candidates (mean age 69.1) and pelvic CT of 34 age-matched controls (mean age 67.9) were used to reconstruct the sacroiliac joints three-dimensionally. The degeneration score of the sacroiliac joints on axial view, as well as the location and volume of vacuum phenomena in the three-dimensionally reconstructed sacroiliac joints, were analyzed.

**Results:**

The total sacroiliac joint degeneration scores were similar in hip osteoarthritis patients and controls but the breakdown of the score revealed that joint space narrowing and vacuum phenomena in the sacroiliac joint increase in hip osteoarthritis, while osteophytes decrease. Three-dimensional reconstruction revealed that the volume of vacuum phenomena in the sacroiliac joint was significantly larger in the hip osteoarthritis group and the vacuum areas were localized in the antero-superior region of the sacroiliac joint.

**Conclusion:**

Our results suggest that hip osteoarthritis and sacroiliac joint degeneration are related, and that with further investigation, the sacroiliac joint may become a new treatment target in hip osteoarthritis.

## Introduction

The sacroiliac (SI) joint is the largest axial joint in the human body, linking the spine and the pelvis, with a mobility of 3° to 8° of nutation [1,2]. Clinically, SI joint disorders may present as low back pain (LBP) or sciatica-like symptoms [3]. In fact, the SI joint is said to be the main cause in 10% to 30% of cases of LBP [4,5]. Recently, reports that SI joint pathology can cause debilitating LBP have increased as various interventions for SI joint pain such as intraarticular injection, nerve branch blocks, ablation or surgical fusion have become more common [6].

The development of diverse imaging techniques has made the clarification of SI joint pathology more plausible than in the past. Degeneration of the SI joint, characterized by joint space narrowing, osteophytes, subchondral sclerosis, cysts and vacuum phenomena, is a common finding in pelvic computed tomography (CT), appearing in 65.1% of adults and increasing with age [7,8]. The exact significance of vacuum phenomena in the SI joint is not known, but we postulated that it could be playing an important role in SI joint degeneration.

Also of note is a connection between SI joint degeneration with adjoining structures, such as the hip or the spine. Degeneration has been reported to increase in the SI joint after spinal fusion or hip arthrodesis [9,10], but the exact mechanism is unknown. The aim of this study was to investigate the relationship between hip osteoarthritis (OA) and SI joint degeneration by examining the SI joints of hip OA patients while focusing on the localization and quantity of vacuum phenomena.

## Materials and Methods

### Collection of pelvic CT data

The institutional ethics committee approval for institution A was obtained in July 2014 (RBMR-1402) and participants provided written informed consent. Preoperative pelvic CT data spanning the SI and hip joints, from the L5 level to the proximal femoral level, of 50 female hip osteoarthritis patients scheduled for hip replacement and taken between April 2014 and January 2016 were obtained and reviewed retrospectively. Male subjects were excluded from the study to eliminate the possible effect of gender. The CT images were taken in a non-load condition on a 16-slice scanner (Aquilion, Canon Medical Systems Europe, Zoetermeer, the Netherlands). Imaging parameters were 320mm field of view, spatial resolution 512 × 512, 120 kV voltage, and 300mA variable current, 1mm slice thickness and reconstruction kernel for bone (FC30). The data was saved in an anonymized DICOM format. After overview of the images, cases with SI joint ankylosis were excluded, as the separation of the ilium from the sacrum for joint observation was impossible. Metallic implants in the pelvic area (such as contralateral hip replacement) were likewise excluded because the metal artifact altered the density of the surrounding bones on CT, making accurate separations of the joints impossible. This resulted in 31 examinations. The AP radiographs of the hip of these patients were examined to determine the Kellgren-Lawrence grade of osteoarthritis.

As a control group, 75 consecutive examinations of anonymized pelvic CTs taken in institution B for unknown indications between 1st February 2017 and 30th April 2017 were obtained. Ethical approval was waived by the institutional review board. The examinations were performed on a 16-slice scanner (Aquilion RXL 16, Canon Medical Systems Europe, Zoetermeer, the Netherlands). Imaging parameters were 320 mm field of view, spatial resolution 512 × 512, 120 kV voltage, and 300 mA variable current, 1 mm slice thickness, with reconstruction kernel for soft tissue (FC03). Of the 75 consecutive examinations, female subjects between ages 50 and 89 were selected as a gender- and age-matched sample. In addition to excluding cases with SI joint ankylosis and metal implantations, pathological conditions of the hip that were obvious on CT such as any joint space narrowing on the coronal view, and bone tumors, were also excluded, as their influence on the SI joint could not be denied. This resulted in 34 examinations with no hip OA (Kellgren-Lawrence grade 0), which were saved in an anonymized DICOM format and evaluated using the method described below.

### Evaluation of SI joint degeneration

SI joint degeneration was evaluated by applying the scoring system described by Backlund et al. (Table 1). The axial view of the SI joint was examined, and according to the grade of degeneration, a maximum of 3 points were allotted to osteophytes, 2 points allotted to joint space narrowing and subchondral sclerosis, and 1 point allotted each to cysts and vacuum phenomena, with reference to the exemplary films described in Backlund’s paper [8]. Because cases with SI joint ankylosis were excluded in the present study, the maximum total scores were 8 per joint and 16 per patient. In addition to comparing the total score between the hip OA group and the control, we compared the scores for each of the five signs of degeneration separately to search for a specific pattern of degeneration. Three orthopedic surgeons, each with more than ten years of clinical experience, examined the pelvic CTs which were blinded for hip pathology and, if any discrepancies in the scores occurred, the final score was determined in consensus.

**Table 1:** Grading for sacroiliac joint degeneration by Backlund et al. [8].

	0	1	2	3

joint space narrowing	normal	focal	general irregular	–
osteophytes	none	minor	prominent	ankylosis*
subchondral sclerosis	none	minor	prominent	–
cysts	none	exist	–	–
vacuum phenomenon	none	exist	–	–

* SI joint ankylosis was excluded from the present study due to technical reasons.

### Three-dimensional reconstruction of SI joint

**Figure 1:**
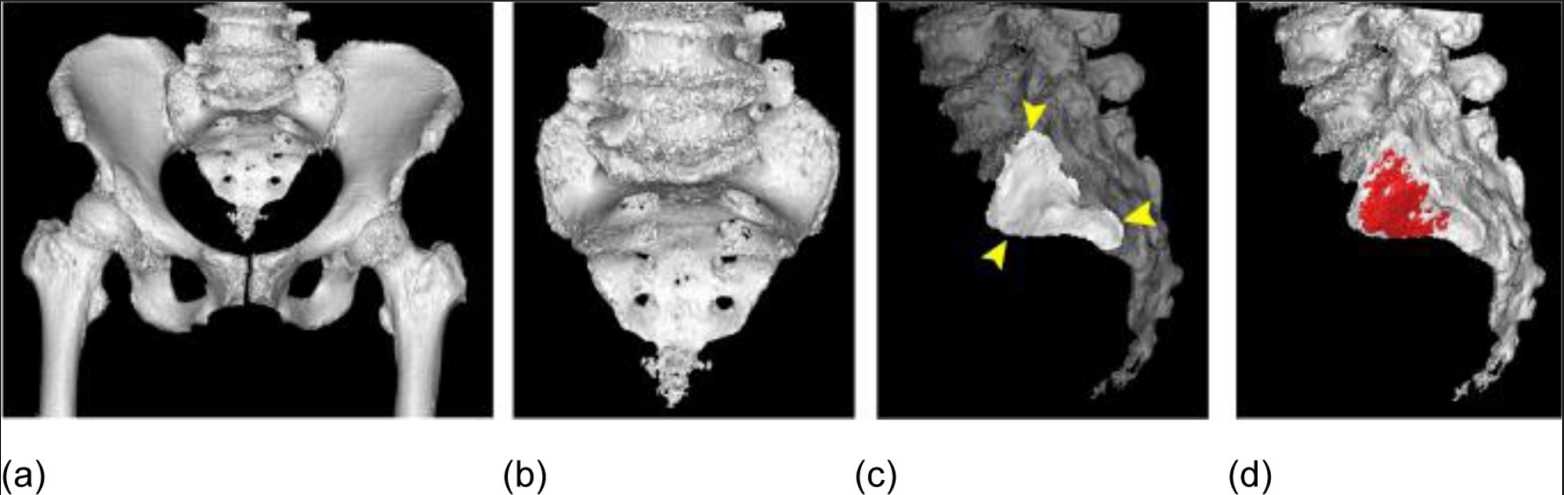
**(a)** Three-dimensional reconstruction and **(b)** separation of the sacrum, **(c)** view of left SI joint surface (area surrounded by yellow bullets), and **(d)** vacuum phenomenon (red) overlaid on the joint surface.

Three-dimensional (3-D) reconstruction of the pelvic bones and vacuum phenomena was achieved using a 3-D reconstruction software package (Mimics® version 20.0, Materialise Inc., Leuven, Belgium). The gaseous area within the SI joints, defined by voxels of less than –300 Hounsfield units as described by Elster and Jensen [16], was extracted separately from the bony structures, reconstructed three-dimensionally and overlaid on the sacroiliac joint surface of the sacrum (Figure 1). The volume of vacuum phenomena was derived using Mimics®, calculated from the number of voxels comprising the vacuum area.

The volume of vacuum phenomena was compared between the hip OA and the control group using the Mann-Whitney U test. The relationship between the SI joint degeneration score and the vacuum volume was analyzed using the Pearson correlation coefficient.

### Statistical Analysis

Significant differences between the two groups were tested using the Mann-Whitney U-test. The correlation between two measures was tested using the Pearson correlation coefficient for interval scales and Spearman’s rank-order correlation for ordinal scales. The data was analyzed using statistical software SPSS ver. 23 (SPSS, Chicago, IL, USA), and p < 0.05 was considered significant.

## Results

### Patient characteristics

The mean age of the hip OA group was 69.1 (standard deviation 7.6) and the control group was 67.9 (standard deviation 8.2, p = 0.688, Mann-Whitney U-test). The Kellgren-Lawrence grades for OA of the 62 hip joints (31 hip OA patients) were 0 in five joints, 1 in thirteen joints, 2 in seven joints, 3 in fourteen joints and 4 in twenty-three joints. All 31 hip OA patients had at least one joint of grade 3 or 4.

### SI joint degeneration score

**Figure 2:**
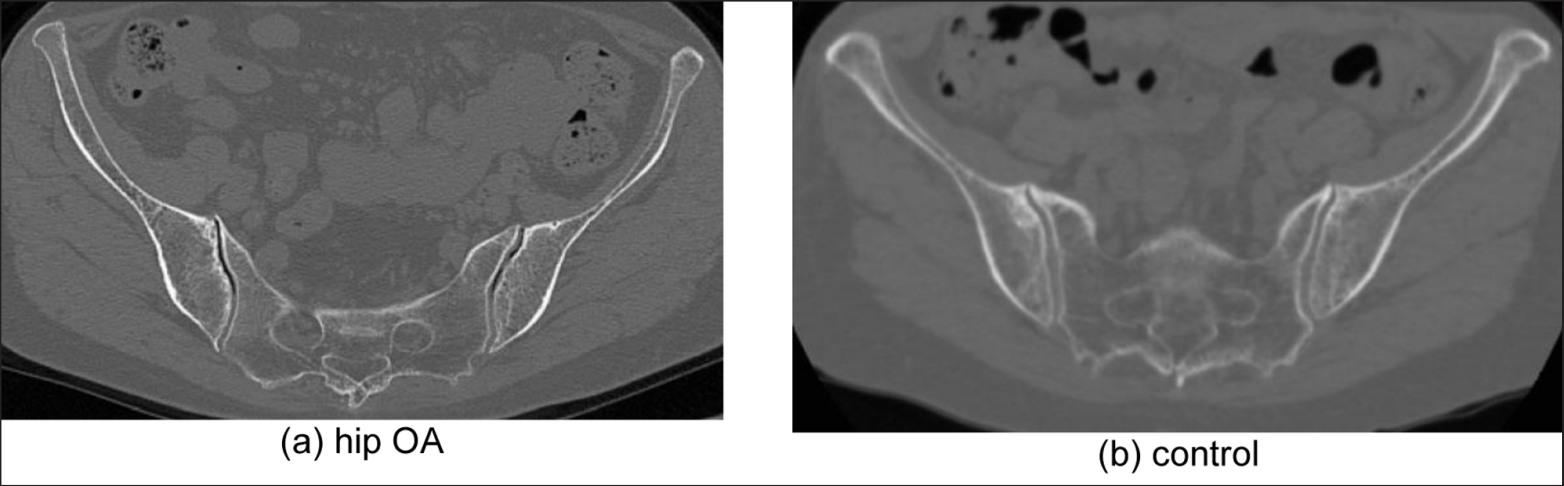
Representative axial views of SI joints exhibiting degenerative changes. **(a)** Vacuum phenomena and joint space narrowing were more distinguishable in hip OA (case no. 23 of Figure 5), while **(b)** the control displayed more pronounced osteophytes.

The total SI joint degeneration score in hip OA patients was similar to that of the control group (p = 0.123). The breakdown of the score revealed that hip OA patients had a significantly higher score for vacuum phenomena and joint space narrowing, and a lower score for osteophytes in the SI joint than those without hip pathology (Table 2, Figure 2). The SI joint degeneration scores of age groups 50–59, 60–69 and 70 and above were not significantly different between hip OA and controls (Table 3).

**Table 2:** Comparison of SI joint degeneration scores and their breakdown in hip OA patients and controls.

SI joint degeneration score (maximum score per patient)	hip OA		control		

	mean	s.d.	mean	s.d.	p

total (16)	12.3	1.9	11.4	2.3	0.123
joint space narrowing* (4)	3.8	0.5	2.8	0.9	<0.001
osteophytes* (4)	2.7	0.7	3.6	0.6	<0.001
subchondral sclerosis (4)	2.6	0.7	2.6	0.8	0.948
Cysts (2)	1.2	0.9	1.1	0.8	0.585
vacuum phenomena* (2)	2.0	0.0	1.3	0.9	<0.001

* p < 0.05, Mann-Whitney U test; OA: osteoarthritis, s.d.: standard deviation.

**Table 3:** Comparison of SI joint degeneration scores divided by age group in hip OA patients and controls.

age group	hip OA			control			

	n	mean	s.d.	n	mean	s.d.	p

50–59	2	12.0	0.0	5	11.6	1.5	0.617
60–69	15	12.3	1.6	15	11.8	2.8	0.613
70+	14	12.2	2.3	14	10.9	2.1	0.126

Mann-Whitney U test; OA: osteoarthritis, s.d.: standard deviation.

### Quantification of vacuum phenomena in the SI joint

**Figure 3:**
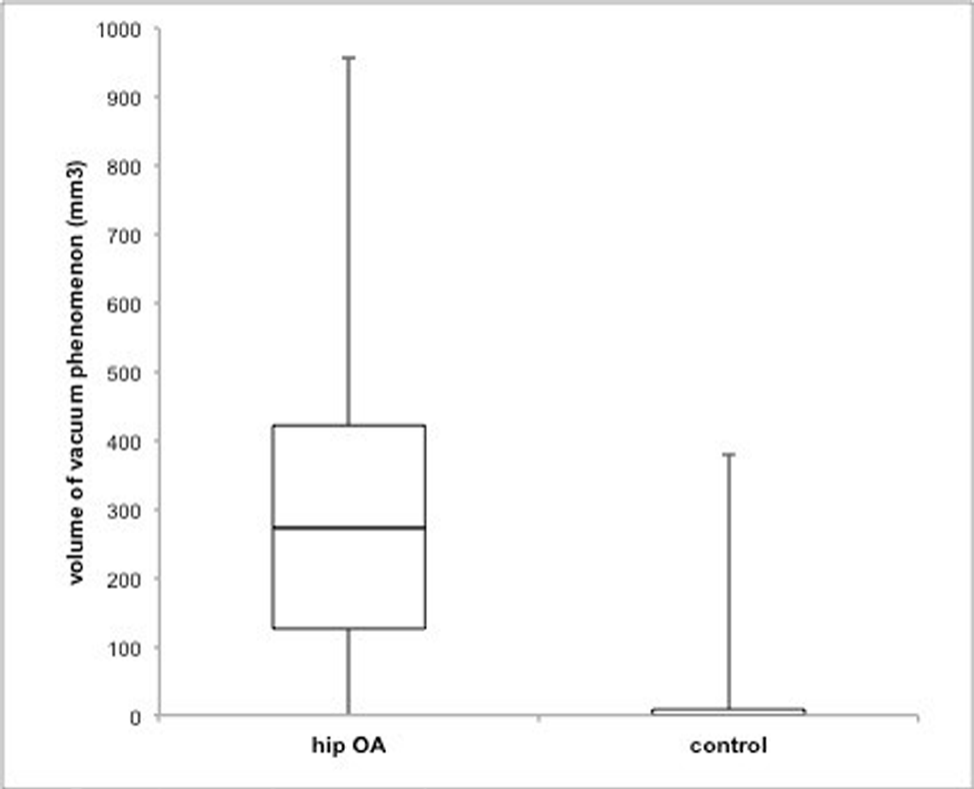
Volume of vacuum phenomena in SI joints of hip OA patients and controls. (p < 0.001, Mann-Whitney U test).

**Figure 4:**
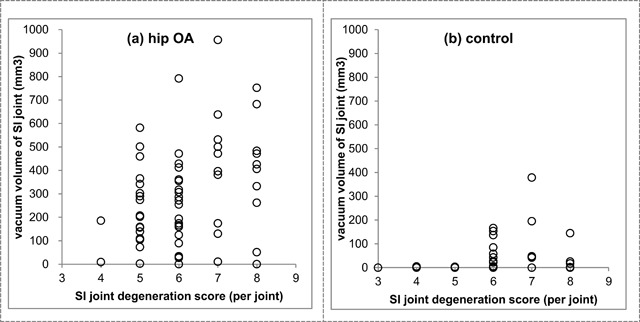
Relationship between degeneration score and vacuum volume of SI joints in **(a)** hip OA group and **(b)** control group.

The volume of vacuum phenomena in the SI joint was significantly larger in the hip OA group (median 273mm^3^, IQR 126-422) than the control group (median 0, IQR 0-9, Figure 3). There was a weak correlation between the SI joint degeneration score and the volume of vacuum phenomena in both the hip OA group (R = 0.333, p = 0.00817, Figure 4a) and the control (R = 0.307, p = 0.0108; Pearson correlation coefficient, Figure 4b).

### Localization of vacuum phenomena in the SI joint

**Figure 5:**
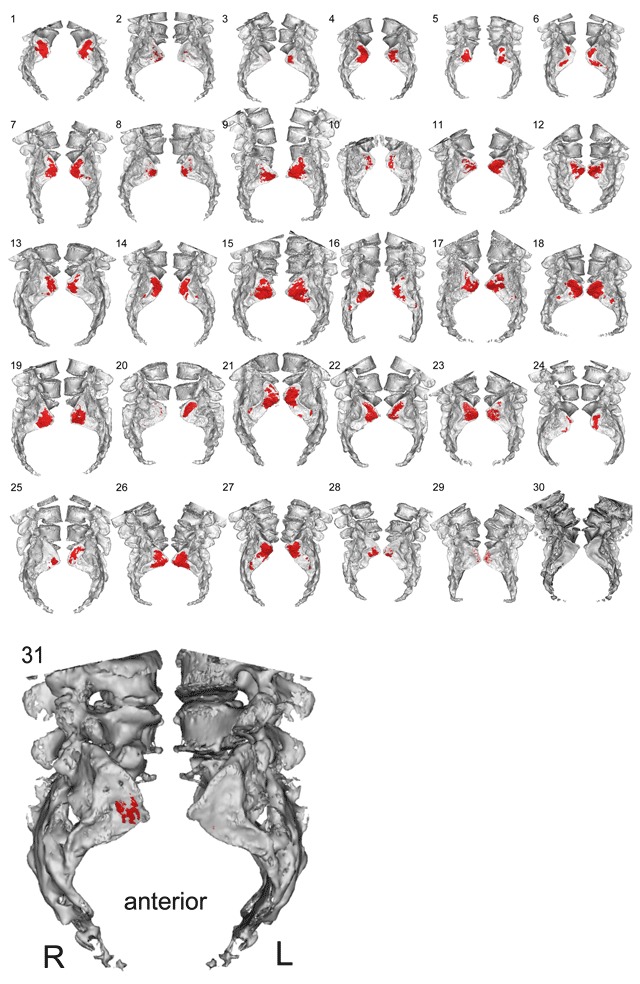
List of the 31 patients with hip OA; many SI joints show significant area of vacuum phenomena (shown in red). Note that the vacuum tends to be in the anteriosuperior (synovial) part rather than the posterior, ligamentous part of the joints. Patient #31’s enlarged view of bilateral 3-D reconstructed SI joint with overlaid vacuum phenomenon.

The 3D reconstruction revealed that the majority of the vacuum phenomena were located in the antero-superior region of the the SI joint (Figure 5).

## Discussion

This study showed that joint space narrowing and vacuum phenomena in the SI joint increase in hip OA, while osteophytes decrease. The biomechanical stress of the SI joint may increase in hip OA through factors such as restriction of the range of motion in the hip, leading to SI joint degeneration. Previous reports of SI joint degeneration are centered on the axial view of CT, and our three-dimensional portrayal of SI joints and vacuum phenomena have never been described. Our results showed that vacuum phenomena are mostly localized in the anterior portion. This area of the SI joint is synovial and more mobile compared to the posterior ligamentous portion [3], and this structural trait may have influenced the distribution of vacuum phenomena.

Past studies have shown that degeneration of the SI joint is more prevalent in the elderly population, but these changes tend to plateau in the higher age groups [7,8]. Our study did not include patients under 50, and the degeneration scores were predictably high and did not display a significant difference between the age groups.

The breakdown of the scores revealed a different pattern of degeneration between the hip OA group and the control group. To be specific, the hip OA group had greater joint space narrowing and vacuum phenomena and smaller osteophytes, and the difference in scores between the two groups was cancelled by addition. In typical diarthrodial joints such as the knee, osteophytes tend to increase as degeneration progresses, as a manifestation of proliferative change. Vacuum phenomena, on the other hand, is uncommon. These distinctions in the pattern of degeneration may be caused by biomechanical forces such as the constant shearing stress distributed on the sacroiliac joint [11]. A possible factor in hip OA that may increase the biomechanical stress on the SI joint is restriction of motion in the hip.

As hip arthrodesis is known to be related to SI joint degeneration [10], limitation in the range of motion of the hip could have similar effects on the SI joint. Our study consisted of end-stage hip OA patients, all with Kellgren-Lawrence grades 3 or 4. As the range of motion of the hip decreases as hip OA progresses [12], the degeneration of the SI joints may have been influenced by stiffness of the hip joint. In other words, hip OA may have caused degeneration of the SI joints to progress. On the other hand, a follow-up study may reveal changes in SI joint degeneration after hip replacement, whereupon joint contractures are usually corrected.

Another factor that may cause biomechanical stress on the SI joint is leg length discrepancy. Kiapour et al. reported, in a finite element analysis model, that a leg length discrepancy of 1 cm increases the load on the SI joint five-fold, and a discrepancy of 3 cm up to 12 times [13]. Though we were unable to analyze its effect on SI joint degeneration in this study because leg length measurements were unavailable, leg length discrepancy is a common manifestation in unilateral hip OA. Future research may reveal whether this could be another contributing factor to SI joint degeneration in hip OA.

Vacuum phenomena was first reported as a hyperlucency on X-ray and CT of spinal compression fractures, occurring as a result of the instability of the fractured vertebrae [14,15,16]. Meanwhile, outside the spine, physiological positions of the joint, or underlying pathologies such as meniscal tear in the knee or condylar fractures in the temporomandibular joint are known to cause vacuum phenomena [17,18]. The existence of vacuum phenomena in the sacroiliac joint has been reported, but this study is the first to quantify them and to examine their relationship to hip OA. There was a significantly higher incidence as well as a larger volume of vacuum phenomena in the SI joint of women with hip OA compared to age-matched controls. The results of our study lead us to believe that SI joint degeneration is a frequent comorbidity in hip OA.

Osteophytes, which were less frequently observed in hip OA patients in this study, are generally involved in stabilization of a degenerating joint [19,20]. Accordingly, our results lead us to consider the possibility that vacuum phenomena in the SI joint is an indication of decreased joint stabilization mechanisms in the course of degeneration.

The biggest limitation of this study is that the clinical data such as SI joint pain and range of motion of the hip was unavailable for analysis. Also, the control group and the hip OA group had different reconstruction kernels, therefore altered Hounsfield units may have caused false positive vacuum phenomena. As the voltage and the CT scanner are two known factors that can alter the Hounsfield units [21,22], we utilized examinations taken in conditions as similar as possible. The small number of subjects and lack of male subjects are some other limitations, as well as the absence of follow-up studies. Lastly, the effects of leg length discrepancy and spinal pathology were not assessed.

## Conclusion

In this study there was a significantly higher incidence as well as a larger volume of vacuum phenomena in the SI joint of women with hip OA compared to age-matched controls, whereas osteophytes showed the inverse relationship. The biomechanical stress of the SI joint may increase in hip OA through restriction of the range of motion in the hip, leading to SI joint degeneration. Further research is needed to clarify this hypothesis as well as the clinical significance of SI joint degeneration and vacuum phenomena in hip OA.
